# Prognostic Impact of KRAS-TP53 Co-Mutations in Patients with Early-Stage Non-Small Cell Lung Cancer: A Single-Center Retrospective Study

**DOI:** 10.3390/jcm14145135

**Published:** 2025-07-19

**Authors:** Lucia Motta, Francesca Molinari, Jana Pankovics, Benjamin Pedrazzini, Alexandra Valera, Samantha Epistolio, Luca Giudici, Stefania Freguia, Miriam Patella, Martina Imbimbo, Giovanna Schiavone, Milo Frattini, Patrizia Froesch

**Affiliations:** 1 Oncological Institute of Southern Switzerland (IOSI), Ente Ospedaliero Cantonale (EOC), 6500 Bellinzona, Switzerland; lucia.motta@humanitascatania.it (L.M.); jana.pankovics@eoc.ch (J.P.); benjamin.pedrazzini@eoc.ch (B.P.); martina.imbimbo@eoc.ch (M.I.); giovanna.schiavone@eoc.ch (G.S.); 2 Institute of Pathology, Ente Ospedaliero Cantonale (EOC), 6900 Locarno, Switzerland; francesca.molinari@eoc.ch (F.M.); alexandra.valera@eoc.ch (A.V.); samantha.epistolio@eoc.ch (S.E.); luca.giudici@eoc.ch (L.G.); stefania.freguia@eoc.ch (S.F.); milo.frattini@eoc.ch (M.F.); 3 Thoracic Surgery, Ente Ospedaliero Cantonale (EOC), 6500 Bellinzona, Switzerland; miriam.patella@eoc.ch

**Keywords:** lung adenocarcinoma, early-stage, KRAS, TP53, STK11, prognosis, survival

## Abstract

**Background/Objectives**: The clinical value of *KRAS* mutations in lung adenocarcinoma, alone or in combination with other mutations, has been assessed especially in advanced stages. This study evaluates how *KRAS* and the presence of co-mutations could affect survival in early-stage lung. **Methods**: We analyzed a real-world cohort including all staged NSCLC patients diagnosed and treated from 2018 to 2022 at our Institute with availability of NGS molecular data. Statistical analyses were made using log-rank test, the two-tailed Fisher’s exact test and Kaplan-Meier survival curves. **Results**: *KRAS* mutations were observed in 179/464 cases (38.6%). The majority of *KRAS* co-mutations were in *TP53* (74%) and *STK11* (14.3%) genes. *KRAS*+*TP53* co-mutations were more frequent compared to *KRAS*-only tumors in stage IV NSCLC (*p* = 0.01). In early stage and locally advanced cases (stage I-III), better prognosis was associated to *KRAS*-only mutated NSCLC and to *KRAS*+*STK11* mutated cases compared to *KRAS*+*TP53* (*p* = 0.008). In particular, patients carrying *KRAS*+*TP53* in stage I and II displayed a shorter survival, similar to patients diagnosed at stage III. **Conclusions**: Routine NGS provides important information for potential actionable mutations but also for the prognostic and predictive role of the presence of co-occurring mutations. In particular, the presence of *KRAS*+*TP53* in stage I and II NSCLC may be considered an unfavorable prognostic marker possibly leading to adapt the perioperative chemo-immunotherapy.

## 1. Introduction

Lung cancer is the leading cause of cancer-related deaths. In 2023, it is estimated that there were more than 235,000 new cases of lung cancer in USA [1]. In last years, the widespread use of molecular analyses has greatly supported treatment decision, especially for adenocarcinoma (AC), the most common histologic subtype [2]. Next-Generation Sequencing (NGS) multigene panels to assess ESCAT (ESMO Scale for Clinical Actionability of molecular Targets) level I alterations (in the *EGFR* and *BRAF* genes, for the presence of KRAS p.G12C mutation and in the fusions involving *ALK*, *ROS*, *MET* and *RET* genes) are recommended for daily practice [3].

Larger NGS panels are highly recommended to be performed in clinical research centers in the context of clinical research and in order to increase access to innovative drugs [4,5]. In addition, *HER2* mutation and other gene alterations (e.g., *NRG1* fusion), are also recommended to be routinely evaluated as targeted drugs have been recently approved [6].

The growing trend in the use of NGS in patients with lung AC revealed how other genes (e.g., *KEAP1*, *STK11*, *TP53*) might have a clinical role, even if no specific targeted drugs have been developed or approved for them [7,8].

*KRAS* mutations are identified in about 30% of lung AC [3] and frequently occur at exon 2 (in the hotspots located at codons 12 and 13), with the most common change represented by the substitution of glycine for cysteine at position 12 (p.G12C) (39%), followed by the p.G12V (21%) and the p.G12D (17%) [9]. *KRAS* alterations have been associated with Caucasian ethnicity, female sex, AC histology and a history of smoking. The mutant KRAS protein has long been considered “undruggable” until the identification of specific KRAS p.G12C inhibitors like sotorasib or adagrasib [10,11]. Based on encouraging data from clinical trials showing a benefit in terms of progression free survival (PFS) and safety profile, these two first-in-class drugs in recent years (from 2022) received conditional authorization and accelerated approval from the US Food and Drug Administration (FDA) and the European Medicines Agency (EMA), respectively [12,13], for patients with advanced lung AC carrying the KRAS p.G12C after first-line treatment with immune checkpoint inhibitors (ICIs), with or without platinum-based chemotherapy [9,14,15].

Even though the prognostic value of *KRAS* mutation in lung AC has not yet been defined, many retrospective studies exploring the role of these mutations have been published [16,17,18,19,20]. Many recent clinical studies have pointed out a possible impact on clinical outcome of the interaction of *KRAS* mutations with other alterations [14,21,22,23]. For example, co-alterations of *KRAS*+*TP53* or *STK11* genes seem to have a negative prognostic impact based on results from different retrospective trials [14,20,24]. Another important aspect is the immune profile of these patients. Tumors with a *KRAS*+*TP53* co-mutation generally exhibit significant upregulation of PD-L1 expression, whereas those with a *KRAS*+*STK11* co-mutation are frequently negative for PD-L1 expression. Therefore, in the future, knowledge of gene alterations concomitant to *KRAS* mutations could guide the use of therapies, especially ICIs [24,25].

Despite significant progress in the molecular characterization of NSCLC, stratifying patients based on their molecular profiles remains a major clinical challenge. The biological complexity of the tumor, combined with the frequent presence of co-mutations and intra-tumoral heterogeneity, makes it difficult to predict clinical behavior and response to targeted treatments. For instance, while *KRAS* mutations are among the most common, their prognosis varies significantly depending on the concomitant presence of other genetic alterations, such as *TP53* or *STK11*, which impact therapy response and clinical outcomes [25]. This variability highlights the need to improve the interpretation of molecular data to optimize personalized therapeutic strategies and increase clinical success rates.

The majority of the studies published to date focused on analyzing the effect of the molecular pattern in metastatic lung AC (stage IV disease), while little is known about early stages [26,27,28,29].

Early stages present better prognosis than advanced NSCLC, being less severe, and are managed with different treatments. Therapies available for advanced cases are not administered in early stage cases as, in general, the standard therapeutic approach in these patients is surgery. However, for some stage I patients, surgery is not enough with nearly 45–65% of cases relapsing after 5 years [30]. The reason for this relapse has not been clearly defined and, to date, few studies focused on this topic. One paper by Rao and colleagues describes how presence of *KRAS* mutation in early stages patients associates with relapse in 1 year time after resection. The mutation frequency of *KRAS* was significantly higher in the rapid relapse group (*p* = 0.008) [30]. Despite all the data described before, to date there is still a lack of knowledge in the field of markers able to predict survival and relapse in early stages.

To better clarify this aspect, we conducted a retrospective real-world analysis of all consecutive lung AC patients, therefore including all the stages, who were followed in a single center, the Oncology Institute of Southern Switzerland (IOSI). Our main objective was to unveil correlations between molecular characteristics and survival in patients with *KRAS*-mutant lung AC; in particular, we focused our attention on early stage cancers, to better understand the influence of molecular analyses in this setting. Above all, we explored the clinical role of *KRAS* and *TP53* co-mutations as prognostic factor.

## 2. Materials and Methods

### 2.1. Patient Population and Study Design

We retrospectively analyzed the medical records of all consecutive patients first diagnosed with lung AC, regardless of the stage, from the first of January 2018 to 31 August 2022. The study was conducted at IOSI and all patients received a treatment based on the stage of the tumor, which was decided after a multidisciplinary discussion and in accordance with the recommendations of the European guidelines [1,2].

For each patient, we collected clinical pathological data from electronic medical records, including gender, age, Eastern Cooperative Oncology Group performance status (ECOG PS) at the time of diagnosis, body mass index (BMI), smoking history (classified as never, former, or current smokers), laboratory values, tumor histology, molecular analysis, tumor staging at the time of diagnosis according to the eighth edition of the American Joint Committee on Cancer [31], sites of metastatic disease, treatments received, and outcomes (date of progression, death, or last follow-up).

This study was approved by the local Ethics Committee (BASEC: 2023-01209; Rif. CE 4402; approval date: 13.07.2023) and all the patients signed the informed consent.

Inclusion criteria were age of 18 years or older at the time of diagnosis; a diagnosis of non squamous non-small cell lung cancer (non-sq NSCLC), availability of clinical data, signed informed consent. Exclusion criteria were unavailability of clinical data, absence of sufficient amount of tissue for performing the molecular characterization, molecular data not evaluable/analyzable, absence of signed informed consent.

### 2.2. Treatment Characteristics

Staging procedures and treatment proposals were developed according to the ESMO Clinical Practice Guidelines and were evaluated within a multidisciplinary team based on tumour staging according to the Union for International Cancer Control UICC TNM eight edition. The decision-making process was carried out by specialists with proved expertise in thoracic oncology and after consultation with the patient. A personalized approach was offered according to tumor-related factors (e.g., molecular pathology, PD-L1 expression) and individual factors (e.g., age, performance status—PS, preexisting comorbidities and patient’s preferences).

All patients with early stage or locally advanced NSCLC were discussed in a multidisciplinary tumour board (MTB). In general, in patients with clinical stages I–III NSCLC, detailed loco-regional staging according to ESMO Clinical Guidelines was performed [32]. Patients with stage I NSCLC underwent surgery with lobectomy or anatomical resection combined with lymph node dissection. Stage II patients underwent surgery (lobectomy or anatomical segmentectomy resection with lymph node dissection). Adjuvant platinum-based chemotherapy was offered to patients with resected stage II and III NSCLC. Patients with resectable Stage III NSCLC received surgery (lobectomy or pneumonectomy with lymph node dissection) and (neo-) adjuvant platinum-based chemotherapy whereas non-resectable Stage IIIB were treated with concurrent chemoradiotherapy with platinum-based doublet, followed by maintenance durvalumab for one year. Generally, systemic therapy was offered to all stage IV patients with PS 0–2. Patients with a PD-L1 tumor proportion score (TPS) ≥ 50% received single-agent immunotherapy as first-line treatment. Patients with a PD-L1 TPS of 1% to 49% received immunotherapy plus platinum-based chemotherapy. For patients with non-sq NSCLC harbouring certain driver alterations (such as in the *EGFR*, *ALK*, *ROS1*, *BRAF*, *RET*, *MET*, or *NTRK* genes), first-line treatment consisted of targeted agents based on the mutation type and the availability (SwissMedic approval) [33].

### 2.3. Tumor Analysis

The molecular characterization of all samples was conducted at the Institute of Pathology EOC in Locarno, Switzerland. Initially, NGS was used to identify point mutations and small insertions or deletions, while fluorescence in situ hybridization (FISH) was applied for gene fusions. However, starting from 2020, NGS has also been used to detect gene fusions. Genomic DNA extraction was performed using the QIAamp DNA Formalin Fixed and Paraffin Embedded (FFPE) Tissue kit (Qiagen, Chatsworth, CA, USA) on three 8 µm thick FFPE sections. The extracted DNA underwent NGS using the Ion Torrent S5XL platform with the commercially available Ion AmpliSeq Colon and Lung Cancer Panel v2 (CLv2) (Thermo Fisher, Waltham, MA, USA). The CLv2 panel provides information on the mutational status of 22 genes frequently mutated in lung AC. FISH analysis was performed on FFPE tissue sections following established criteria [16,18]. Immunohistochemical evaluation of PD-L1 protein expression was conducted using an automated instrument and the SP263 monoclonal rabbit anti-human antibody [19]. Starting from 2020, the Archer FusionPlex Lung NGS Panel (Archer, Boulder, CO, USA) was applied for gene fusion detection. In this case total RNA was extracted using the ReliaPrep FFPE Total RNA Miniprep System (Promega, Madison, WI, USA) and RNA was analyzed through an anchored Multiplex PCR and NGS. The Archer FusionPlex Lung NGS Panel focuses on detecting gene fusions, including exon skipping, in 14 genes, including *ALK*, *ROS1*, *RET*, *MET*, *NTRK1-2-3*. A case was defined as mutated in a particular gene if the Variant allele frequency (VAF) in a tissue sample was ≥5% and if the method had a good coverage result (>500 for all the regions).

### 2.4. Statistical Analyses

Descriptive statistics were used to summarize patient demographics and tumor characteristics, including measures such as mean, median, and proportions. Differences in continuous variables were assessed using the two-tailed Fisher’s exact test. Overall Survival (OS) was measured from the date of biopsy-proven diagnosis to the date of death. For patients who were still alive at the end of the study, their data were censored at the time of their last available follow-up. Kaplan-Meier survival curves were generated to estimate OS and group comparisons were conducted using the log-rank test. Statistical significance was set at *p* < 0.05. All analyses were conducted using GraphPad Prism version 9.5.1.

## 3. Results

### 3.1. Description of the Entire Cohort

We identified 464 patients with non-sqNSCLC consecutively diagnosed in our Institution from January 2018 to August 2022. The characteristics of all the study population are reported in Table 1: Two hundred and fifty seven were men (55.4%), the median patient age at diagnosis was 73 years (range, 30–99 years). Concerning smoking history, 367 patients (79.1%) were current or former smokers. The majority of cases were diagnosed with lung AC (*n* = 456; 98.3%). Stage IV cases have been diagnosed in 259 patients (55.8%) with a 16.2% harboring brain metastases. PD-L1 expression was >50% in 114 patients (24.6%). After a median follow-up of 30.47 months, with 229 deaths recorded, the population’s median OS (mOS) was 17.5 months. Dividing the population into stages, we observed the following mOS values: for stage I and II mOS has not been reached, for stage III and IV the mOS was 32.7 and 11.8 months, respectively (*p* < 0.0001; Figure S1). At molecular level, thanks to NGS analysis, we found alterations in different potentially targetable genes (Figure S2): *KRAS* (*n* = 179; 38.6%), *EGFR* (*n* = 64; 14.2%), *BRAF* (*n* = 15; 3.6%), *HER2* (*n* = 2; 0.4%), *ALK* (*n* = 10; 2.1%), *ROS1* (*n* = 1; 0.2%), *RET* (*n* = 1; 0.2%), *NTRK* (*n* = 1; 0.2%), *MET* exon skipping (*n* = 15; 4.7%). Other genomic alterations have been identified: 203 (43.7%) patients had a mutation in the *TP53* gene, 17 (3.7%) in *STK11* and 42 (9%) in other genes including *FGFR2*, *SMAD4*, *ERBB4*, *PTEN*, *CTNNB1*, *PIK3CA* and *FBXW7*.

### 3.2. Impact of KRAS Mutational Status

The clinical and molecular characteristics of *KRAS* mutant are described in Table 2: 98 were men (54.7%), the median patient age at diagnosis was 70 years (range, 45–86 years) and 160 patients (89.2%) were current or former smokers. Most patients were diagnosed with lung AC (*n* = 177; 98.9%) and 100 (55.9%) had clinical stage IV disease at diagnosis, with a 14% incidence of brain metastases. PD-L1 expression levels were >50% in 55 patients (30.7%). The main *KRAS* mutations detected were p.G12C (47.5%), p.G12V (19%) and p.G12D (11.7%) changes.

To assess correlations among the various clinical-molecular characteristics, we used the two-tailed Fisher’s exact test and we observed a statistically significant association between having a *KRAS* mutation and a positive smoking history (*p* < 0.0001). Instead, there were no associations between the presence of *KRAS* mutations and gender, BMI, age at diagnosis, brain metastases, or TNM stage. Finally, there was a statistically significant association between the presence of *KRAS* mutations and the absence of *EGFR* (*p* < 0.0001) and *BRAF* (*p* = 0.004) mutations, and *ALK* rearrangements (*p* = 0.008). Therefore, our data confirmed the mutual exclusivity among these molecular alterations.

**Figure 1:**
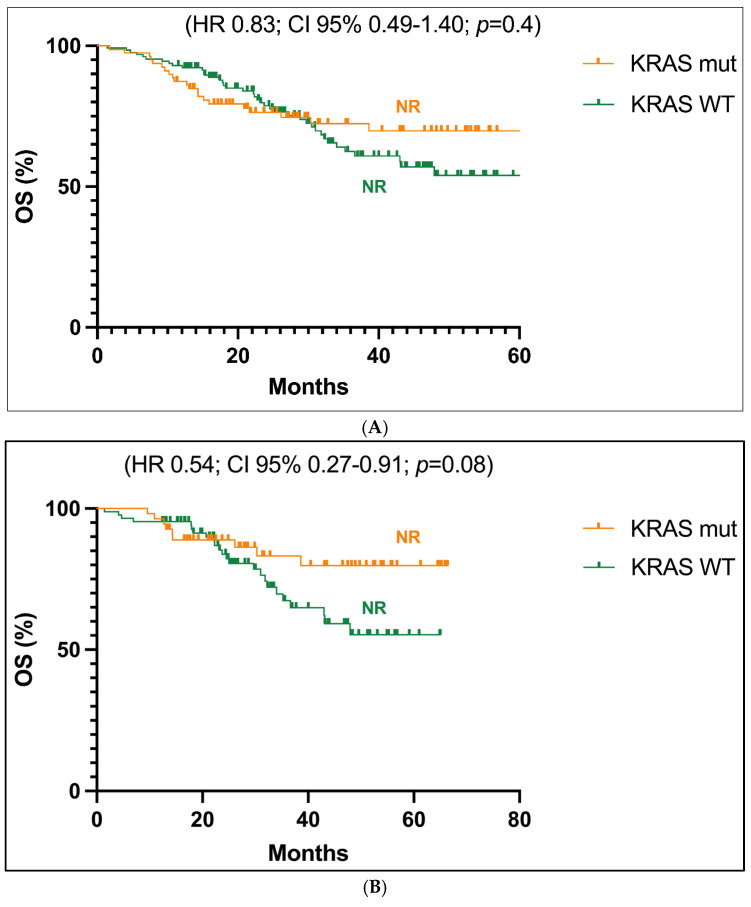
(**A**). Overall survival (OS) analysis of the population in stages I-III with *KRAS*-mutated (*KRAS* mut) versus non-mutated (*KRAS* WT) status. (**B**). Overall survival (OS) analysis of the population in stages I-II with *KRAS*-mutated (*KRAS* mut) versus non-mutated (*KRAS* WT) status. Abbreviations: CI, confidence interval; HR, hazard ratio; NR, not reached.

Focusing on stages I-III, the Kaplan-Meier survival curves showed a not reached value of mOS in both patients with *KRAS* mutant tumors and with a *KRAS* WT sequence, with no significant statistical difference between these two groups (HR 0.83; CI 95% 0.49–1.40; *p* = 0.4; Figure 1A). Similar results were obtained when only early stages (stage I and II) were taken into account, although a trend in favour of better follow-up for *KRAS* mutant with respect to *KRAS* WT cases can be observed, with a HR value of 0.54 (Cl 95% 0.27–0.91; *p* = 0.08) (Figure 1B).

### 3.3. Impact of Co-Occurring Mutation

Table 3 reports the number of cases with *KRAS* WT, *KRAS*-only mutations or *KRAS*+TP53 co-mutations subdivided according to the stages. The number of cases with *KRAS* mutations and *KRAS* WT are well balanced in all the stages.

As described in Table 2, we have also identified concomitant mutations in 43% of cases, involving genes such as *TP53* (74%), *STK11* (14.3%), and others (24.7%; *MET*, *BRAF*, *FGFR2*, *SMAD4*, *ERBB4*, *PTEN*, *CTNNB1*, *PIK3CA*, *FBXW7*).

Among these molecular alterations, the only statistically significant correlations were those between *KRAS* and *STK11* and *KRAS* and *TP53*. As regards the first couple, among 17 patients with *STK11* mutations, 11 had concurrent *KRAS* mutations (*p* = 0.03). Concerning the second couple, our data revealed a statistically significant higher occurrence of *TP53* mutations in patients with *KRAS* WT tumors compared to those with *KRAS* mutation (31.17% vs. 12.34%, *p* < 0.0001). *TP53* co-mutation also appears to be correlated with the disease stage. The probability of an early stage diagnosis is higher in individuals without co-mutations (*KRAS*-only). Fisher’s exact test revealed that in patients with *KRAS*+*TP53* co-mutations, compared to those with *KRAS*-only tumors, there were a greater likelihood of being diagnosed at stage IV rather than stage I, II and III (*p* = 0.01). Interestingly, in our cohort, only 5.4% (3 patients out of 55) of patients with *KRAS* and *TP53* co-mutations has stage I at diagnosis (Table 3).

**Figure 2:**
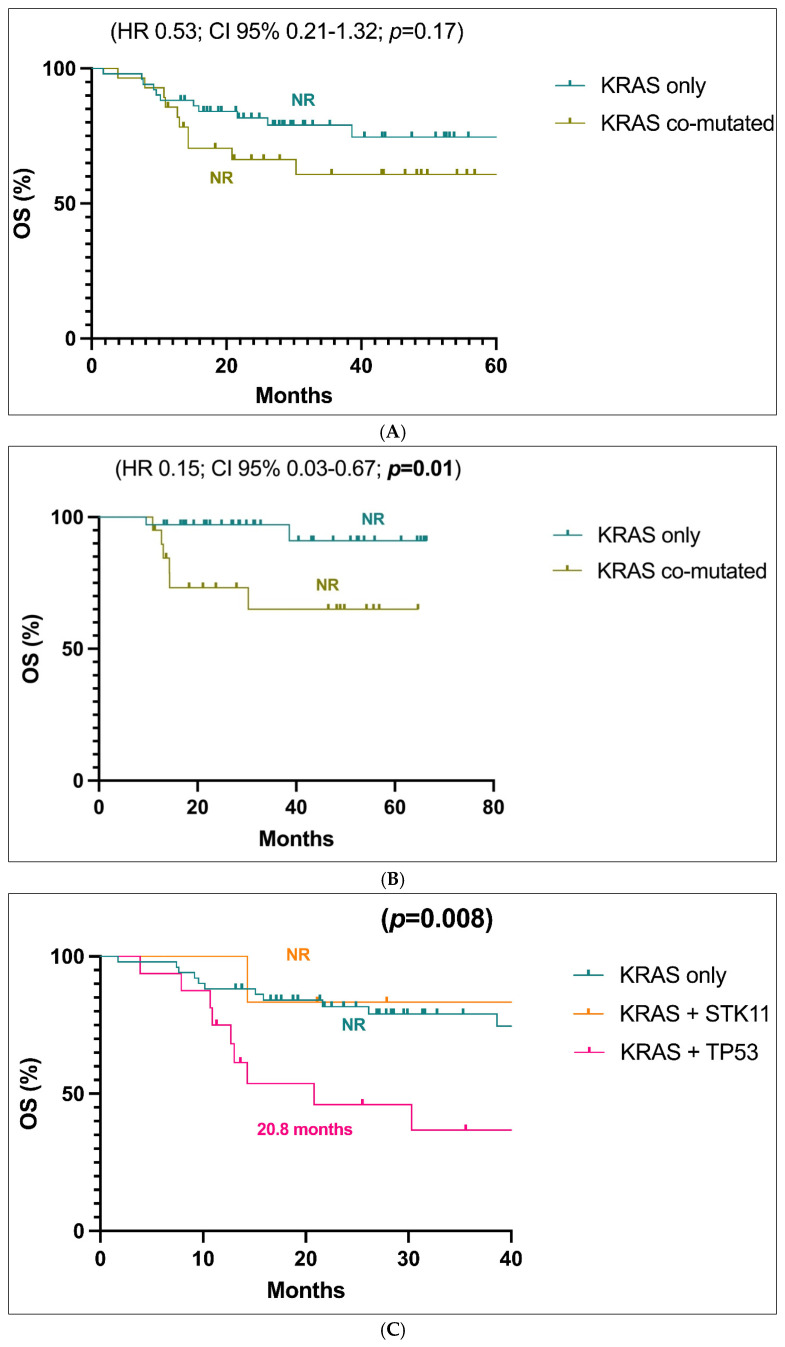

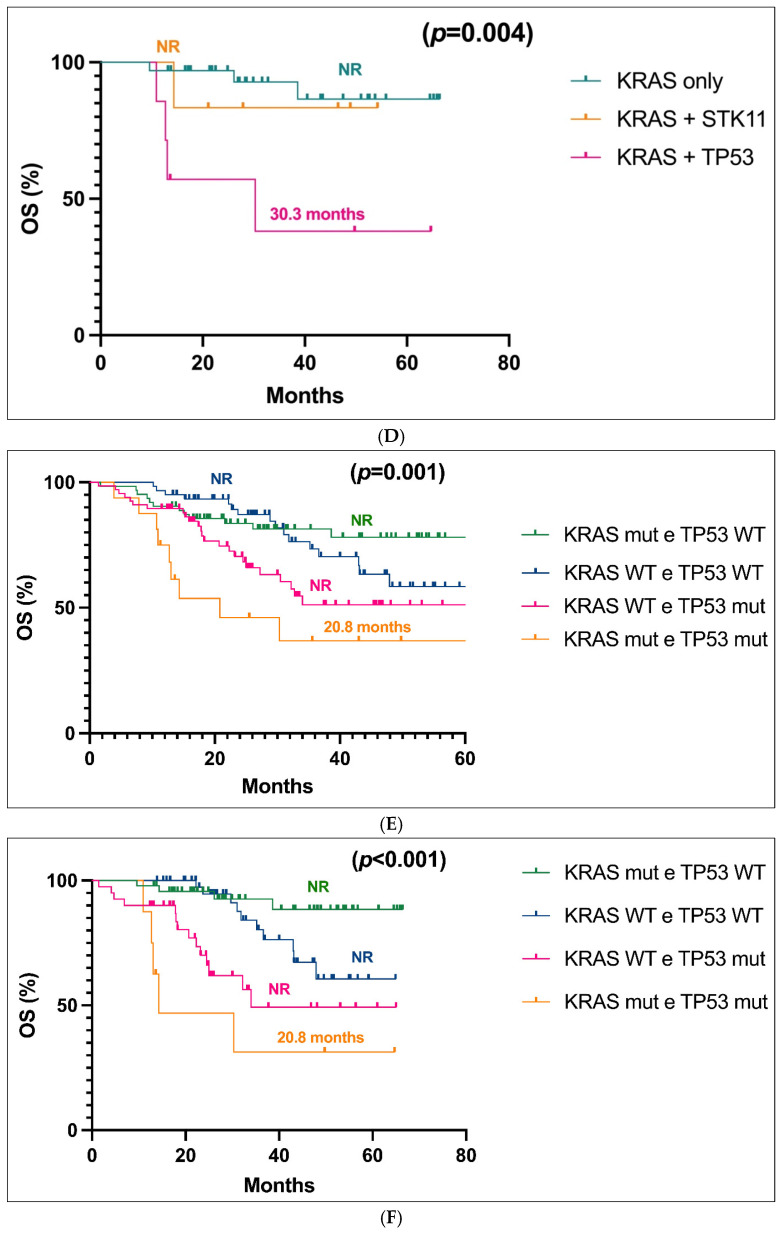
(**A**). Overall survival (OS) analysis of the population in stages I-III with *KRAS*-only versus *KRAS* co-mutated tumors. (**B**). Overall survival (OS) analysis of the population in stages I-II with *KRAS*-only versus *KRAS* co-mutated tumors. (**C**). Overall survival (OS) analysis of the population in stages I-III with *KRAS*-only versus *KRAS*+*STK11* and *KRAS*+*TP53* mutated tumors. (**D**). Overall survival (OS) analysis of the population in stages I-II with *KRAS*-only versus *KRAS*+*STK11* and *KRAS*+*TP53* mutated tumors. (**E**). Overall survival (OS) analysis of the stages I-III population with *KRAS*+*TP53* mutations versus only *KRAS*, only *TP53* and *KRAS* and *TP53* WT. (**F**). Overall survival (OS) analysis of the stages I-II population with *KRAS*+*TP53* mutations versus only *KRAS*, only *TP53* and *KRAS* and *TP53* WT. Abbreviations: CI, confidence interval; HR, hazard ratio; NR, not reached.

The survival analyses did not reveal a statistically significant difference between stages I-III patients with *KRAS*-only tumors versus those with *KRAS* co-mutations, although a trend in favour of better prognosis for *KRAS*-only patients can be observed (Figure 2A). Both populations exhibited a not reached value of mOS (HR 0.53; CI 95% 0.21–1.32; *p* = 0.17). The trend in favour of better prognosis for *KRAS* only patients in stages I-III became significant when only stages I and II were evaluated: in this cases, the HR value is 0.15 (Cl 95% 0.03–0.67; *p* = 0.01) (Figure 2B). To assess whether this analysis was adequately powered, we performed a post hoc power analysis. Assuming a two-sided log-rank test with α = 0.05, a total sample size of 44 patients (36 KRAS-only vs. 8 KRAS+TP53), and approximately 15 observed events, the estimated power to detect a hazard ratio (HR) of 0.5 was approximately 40%.

Upon analyzing specific co-mutations, we found that patients with co-mutated *KRAS*+*TP53* tumors had a significantly shorter mOS, while those with *KRAS*+*STK11* co-mutations had comparable mOS to *KRAS*-only patients whether stages I-III (*p* = 0.008, Figure 2C) or stage I-II were analyzed (*p* = 0.004, Figure 2D).

In light of the obtained results, we analyzed OS of patients with stage I-III or only I-II carrying both *KRAS*+*TP53* mutations, compared to those with only *KRAS* or only *TP53* alterations and those with *KRAS* and *TP53* WT status (Figure 2E and Figure 2F, respectively). The four subgroups displayed a statistically significant different OS in both groups of patients (*p* = 0.001, Figure 2E; *p* < 0.001, Figure 2F), with patients carrying a simultaneous *KRAS*+*TP53* mutations experiencing the worst OS (20.8 months) (Figure 2E,F).

**Figure 3:**
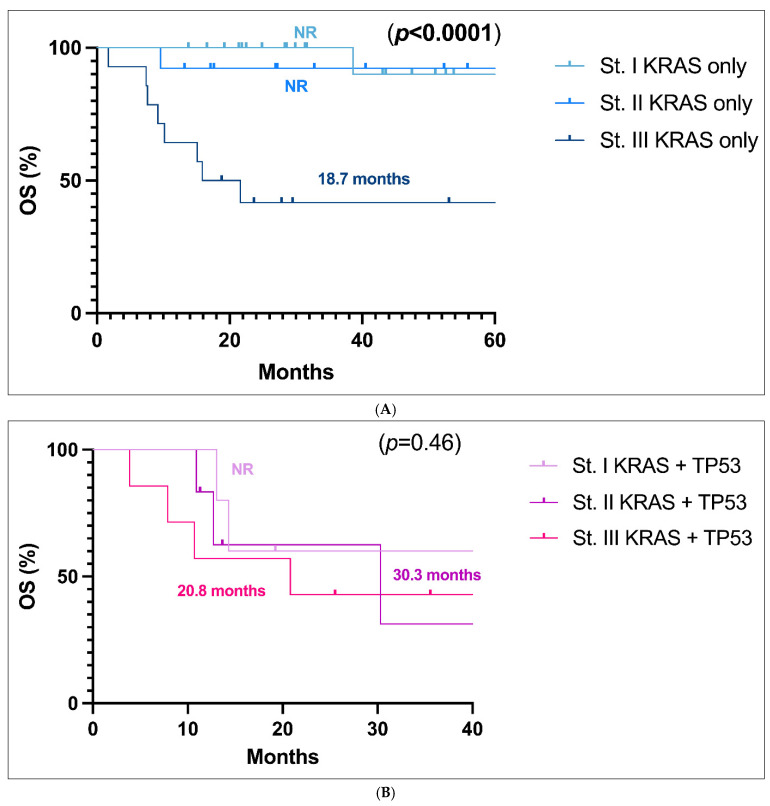
(**A**). Overall survival (OS) analysis of the *KRAS* only population in stage I, II, and III respectively. (**B**). Overall survival (OS) analysis of the *KRAS* and *TP53* co-mutated population in stage I, II, and III respectively. Abbreviations: CI, confidence interval; HR, hazard ratio; NR, not reached.

Finally, we compared the OS of *KRAS*-only (Figure 3A) patients and *KRAS*+*TP53* co-mutated patients (Figure 3B) in stage I vs. II vs. III respectively. In patients with only a *KRAS* mutation, stage I and II patients have not yet reached a median OS, whereas in stage III, the mOS was 18.7 months, with a significant difference between stage III vs. I and II (*p* < 0.0001), while curves of stage I and stage II for *KRAS*-only patients are substantially superimposable (Figure 3A). When *KRAS*+*TP53* co-mutated cases were taken into account, on the contrary, the curves of stages I, II and III were all superimposable, with mOS not reached in stage I, 30.3 months in stage II, and 20.8 months in stage III (*p* = 0.46) (Figure 3B).

## 4. Discussion

Here we report a retrospective analysis of a consecutive Swiss population-based ns-NSCLC cohort, subjected to NGS analysis at the time of diagnosis, focusing especially on stage I-III or stage I-II *KRAS* mutant patients.

Multiple previous analyses focused on stage IV cohorts and yielded conflicting evidence regarding the predictive significance of these concurrent alterations [10,12,20]. With access to molecular data from stage I-III patients, our study has the advantage of including all patients diagnosed with ns-NSCLC, enabling us to identify correlations among markers that may be specific to certain stages. We decided to especially focus our attention on early-stages because today little attention has been paid to the effect caused by specific molecular alterations on therapies in early stages, especially in stage I.

Our initial analysis showed that the incidence rates of targetable mutations were generally consistent with the available evidence confirming the representativeness of our cohort. An exception is *KRAS*, which appeared to be more prevalent in our dataset compared to The Cancer Genome Atlas (TCGA) group [13]. We presume that this difference can be attributed to the high rate of smokers in our region, a factor that is directly correlated to the incidence of *KRAS* mutations [9], as also demonstrated in our cohort.

Our analyses, on one hand confirmed the mutual exclusivity of *EGFR*, *KRAS* and *BRAF* mutations and *ALK* rearrangements, and on the other hand pointed out that *TP53* mutations occur more frequently in *KRAS* WT patients and in advanced/metastatic (IV) stages, thus indirectly indicating a particular negative prognostic factor for such a molecular profile.

Then we took into consideration the clinical data and matched them to the molecular ones. At first, as expected, the mOS by stage confirmed that patients outcome worsen when the disease is diagnosed at a more advanced stage compared to an earlier stage, further indicating the consistency of our cohort: our data are in agreement with the practice guidelines for diagnosis, treatment and follow-up of early and locally advanced NSCLC [32].

To better understand the impact of the presence of a *KRAS* mutation, we compared the mOS with and without this alteration of patients with stage I-III or stage I-II. The analysis confirms that the presence of the *KRAS* mutation by itself should not be considered a prognostic factor. On the contrary, it appears that the prognosis should be more significantly influenced by the presence of concurrent mutations. At first, specifically in stages I-II, the occurrence of a mutation limited to *KRAS* (“*KRAS*-only”) identifies a group of cases with better prognosis if compared to patients with *KRAS* co-mutations. This datum is lost when stage III patients are added, although a similar trend can still be observed. However, the role played by co-mutations may not be unique and therefore we evaluated our cohort dividing co-mutated patients into two groups: one with *KRAS*+*TP53* mutations and one with *KRAS*+*STK11* mutations, i.e the two largest subgroups of patients. From this grouping, we obtained a significant difference which is highly noticeable, as we demonstrated that patients with stage I-II carrying both *KRAS*+*TP53* mutations experience a worse outcome if compared to patients with only a *KRAS* mutation or carrying *KRAS*+*STK11* co-mutations. In 2019, La Fleur and colleagues had already analyzed a cohort of patients who underwent surgery for early-stage NSCLC, and they found a worse prognosis in individuals with *TP53* mutations, thus reinforcing the results of our study [14]. These data were also supported by the MSK-IMPACT study, a large-scale research analysis carried out on 1563 lung cancers [34].

The observed adverse prognostic significance of *TP53* mutations in NSCLC is strengthened by a further stratification: patients with only *TP53* alterations had a poorer prognosis compared to those with *KRAS*-only mutations or those with *KRAS* and *TP53* WT status. However, when we compare the mOS of these patients with those having *KRAS*+*TP53* co-mutations, we can observe that the mOS was even worse than the *TP53*-only subgroup, thus hypothesizing a synergistic negative effect of these two alterations.

This concept is ultimately affirmed by the analysis of OS in *KRAS*-only patients and *KRAS*+*TP53* co-mutated patients in stages I, II and III evaluated separately. In *KRAS*-only subjects, we observe a worse OS among stage III patients than patients at stage I and II (the last two groups showing a superimposable value between them). Conversely, in *KRAS*+*TP53* co-mutated patients, both stage I and stage II exhibit OS rates superimposable between them but also similar to those in stage III. Our results may indicate that *KRAS*+*TP53* co-mutated patients at stage I experience a really negative outcome, similar to the one of stage III patients.

Our analyses may therefore have relevant clinical implications. In fact, recent phase III studies have prompted the clinical community to reconsider the treatment approach for patients with early-stage NSCLC. The CheckMate 816, and other recent trials have shown that chemo-immunotherapy provides benefits for patients starting from stage II, although the subgroup analysis has mainly shown greater advantages in stage III patients [21,35,36,37,38,39,40]. Additionally, in the adjuvant setting, the PEARLS and IMPOWER 010 trials have demonstrated benefit in adding immunotherapy to adjuvant chemotherapy for stage II-III patients. Also in this case, the evidence is stronger for the stage III subgroup [22,23]. To date, there is no biomarker which enable to identify patients with early stage I-II NSCLC that could benefit more from a (neo)- adjuvant (chemo)-immunotherapy. Therefore, it remains unclear whether pre- or post-operative chemo-immunotherapy is necessary for all patients with stage II NSCLC. We likely lack sufficient information to determine whether some patients diagnosed at these stages may benefit more from one therapy over another. However, our analysis suggests that the molecular characterization of tumors may help on this point: patients with a *TP53* mutated or a *KRAS*+*TP53* co-mutated tumor seem to have a poor prognosis and maybe they could represent the group of patients which can benefit effectively from chemo-immunotherapy in the neoadjuvant or adjuvant setting in very early stage (stage I). On the other hand, in our retrospective analysis patients with *KRAS*-only tumors exhibit more favourable prognosis suggesting that in this group we may consider a more observational approach and with more data we could potentially reduce pharmacological therapies in the initial stages.

In our study, unlike the *KRAS*+*TP53* double mutant, considerations and analysis regarding *KRAS*+*STK11* co-mutations cannot be extended further due to low number of cases harbouring mutations in both these markers. The low number of these double mutant cases explain also why we cannot confirm the data in literature reporting how *STK11* co-mutation associate to worse outcomes in NSCLC [8,11,13]. Although the observed prognostic impact of *KRAS*+*TP53* co-mutations in stage I–II NSCLC was statistically significant (HR = 0.15, *p* = 0.01), we acknowledge that this analysis was based on a limited number of patients. A post hoc power analysis revealed that our study had approximately 40% power to detect a moderate effect size (HR = 0.5), indicating that the study may be underpowered for detecting smaller differences. Therefore, while our findings suggest a strong prognostic signal, they should be interpreted cautiously and validated in larger, prospective cohorts.

The strength of our study lies in the presence of a homogeneous population, consecutively diagnosed with NGS analysis and treated at the same Institute in accordance with current European guidelines [1,2].

At the same time, the fact that we analyzed a small single-cohort of homogenous population could be a limitation. Indeed, we can underestimate the clinical role of less frequently altered genes. As a consequence, the enlargement of the number of cases could permit to obtain data also for the categories that are not enough represented in this study, such as the *KRAS*+*STK11* double mutant. These data could be relevant especially on the light of the results obtained from the IMpower150 trial. In this trial a relevant conclusion was that cases with *KRAS*/*STK11* co-mutations treated by atezolizumab plus bevacizumab plus chemotherapy had a longer survival then patients treated only by atezolizumab plus chemotherapy or bevacizumab plus chemotherapy [41].

A second limitation of this study is the relatively small number of genes included in the NGS panel that we applied. This panel is a commercially available solution recommended for diagnostic purposes because it represents a good compromise between low costs and an adequate complete characterization of NSCLC. However, this panel does not include some relevant emerging markers, such as *KEAP1*, which therefore cannot be evaluated in our cohort (by considering them as *KRAS*-only mutant cases). Consequently, there was a minority of *KRAS*-only patients that probably should have been considered within the subgroup of *KRAS* co-mutated individuals.

## 5. Conclusions

In conclusion, although obtained in a retrospective study, our analysis shows that routine NGS provides important information not only for potential actionable mutations but also for the prognostic and predictive role of the presence of co-occurring mutations: in particular, it seems that the assessment of *TP53* mutations may be of a pivotal interest even in early stages. This crucial information potentially leads to a molecular reclassification of ns-NSCLC based on the identified alterations and may have important clinical relevance in adapting the treatment with chemo/immunotherapy in the (neo)-adjuvant setting. Future studies including larger cohort and, above all, prospective series must be done to confirm our hypothesis.

## Figures and Tables

**Table 1:** Characteristics of all population.

All Population (*n*= 464)	
	*n* (%)
Sex	
Male	257 (55.4%)
Female	207 (44.6%)
Age at diagnosis [range], years	
	73 [30–99]
Smoking habit	
Current	220 (47.4%)
Former	147 (31.7%)
Never	80 (17.2%)
Unknown	17 (3.7%)
Histology	
Adenocarcinoma	456 (98.3%)
Adeno-squamous carcinoma	8 (1.7%)
Stage at diagnosis	
I	100 (21.6%)
II	41 (8.8%)
III	62 (13.4%)
IV	259 (55.8%)
Brain metastases in stage IV patients	
No	388 (83.6%)
Yes	75 (16.2%)
Unknown	1 (0.2%)
PD-L1 (TPS) expression	
Negative	168 (36.2%)
1–49%	166 (35.8%)
≥50%	114 (24.6%)
Unknown	16 (3.4%)
Gene Alterations	
No	68 (14.66%)
*KRAS*	179 (38.6%)
*MET*	15 (3.2%)
*EGFR*	65 (14%)
*ALK*	10 (2.2%)
*ROS*	1 (0.2%)
*RET*	1 (0.2%)
*TP53*	199 (42.9%)
*STK11*	17 (3.7%)
*HER2*	2 (0.4%)
*BRAF*	17 (3.7%)
*NTRK*	1 (0.2%)
Other *	33 (7.1%)

* *FGFR2*, *SMAD4*, *ERBB4*, *PTEN*, *CTNNB1*, *PIK3CA*, *FBXW7*.

**Table 2:** Characteristics of the *KRAS* mutant population.

*KRAS* Positive Population (*n* = 179)	
	*n* (%)
Sex	
Male	98 (54.7%)
Female	81 (45.3%)
Age at diagnosis [range], years	
	70 [45–86]
Smoking habit	
Current	106 (59.2%)
Former	54 (30.2%)
Never	12 (6.7%)
Unknown	7 (3.9%)
Histology	
Adenocarcinoma	177 (98.9%)
Adeno-squamous carcinoma	2 (1.1%)
Stage at diagnosis	
I	35 (19.6%)
II	20 (11.2%)
III	22 (12.3%)
IV	100 (55.9%)
Brain metastases in stage IV patients	
No	153 (85.5%)
Yes	25 (14%)
Unknown	1 (0.5%)
PD-L1 (TPS) expression	
Negative	59 (33%)
1–49%	61 (34.1%)
≥50%	55 (30.7%)
Unknown	4 (2.2%)
Mutation type	
p.G12C	85 (47.5%)
p.G12V	34 (19%)
p.G12D	21 (11.7%)
p.G12A	9 (5%)
Other *	30 (16.8%)
*KRAS* concomitant alterations	
No	102 (57%)
Yes	77 (43%)
*MET*	5 (6.5%)
*EGFR*	1 (1.3%)
*ALK*	0 (0%)
*ROS*	0 (0%)
*RET*	0 (0%)
*TP53*	55 (74%)
*STK11*	11 (14.3%)
Other **	14 (18.2%)

* p.G12S, G12R, G12F, G13D, G13C, L19F, A146T, Q22K, Q61H, Q61L. ** BRAF, *FGFR2*, *SMAD4*, *ERBB4*, *PTEN*, *CTNNB1*, *PIK3CA*, *FBXW7.*

**Table 3:** *KRAS* wt, *KRAS* mutations and *KRAS*+*TP53* mutations on the basis of tumor stage.

	*KRAS* WT	*KRAS*-Only	*KRAS*+*TP53* Mut
**Stage I**	65/285 (22.8%)	23/102 (22.6%)	3/55 (5.4%)
**Stage II**	21/285 (7.4%)	13/102 (12.7%)	5/55 (9.1%)
**Stage III**	40/285 (14%)	14/102 (13.7%)	6/55 (10.9%)
**Stage IV**	159/285 (55.8%)	52/102 (51%)	41/55 (74.6%)

## Data Availability

The datasets used and analyzed during the current study are available from the corresponding author upon reasonable request. The data are not publicly available due to institutional policy.

## Supplementary Materials

The following supporting information can be downloaded at: https://www.mdpi.com/article/10.3390/jcm14145135/s1, Figure S1: Overall survival analysis of the entire population divided by stages; Figure S2: Driver molecular characteristics of all populations.

## Abbreviations

The following abbreviations are used in this manuscript:

AC	Adenocarcinoma
BMI	Body mass index
CI	Confidence interval
ECOG PS	Eastern Cooperative Oncology Group performance status
EMA	European Medicines Agency
ESCAT	ESMO Scale for Clinical Actionability of molecular Targets
ESMO	European society for medical oncology.
FDA	US Food and drug administration
FFPE	Formalin-fixed paraffin embedded
FISH	Fluoresence in situ hybridization
HR	Hazard ratio
ICIs	Immune checkpoint inhibitors
IOSI	Oncology institute of Southern Switzerland
MTB	Multidisciplinary tumour board
NGS	Next-generation sequencing
Non-sq NSCLC	Non-squamous Non-Small Cell Lung Cancer
NR	Not-reached
NSCLC	Non-Small Cell Lung Cancer
OS	Overall survival
PFS	Progression-free survival
PS	Performance status
VAF	Variant allele frequencies
