# Supplementary material for: Prognostic Impact of KRAS-TP53 Co-Mutations in Patients with Early-Stage Non-Small Cell Lung Cancer: A Single-Center Retrospective Study
Source: J Clin Med. 2025 Jul 19;14(14):5135. doi: 10.3390/jcm14145135 (PMC12295068; doi:10.3390/jcm14145135)

Figure S1. Overall survival analysis of the entire population divided by stages.

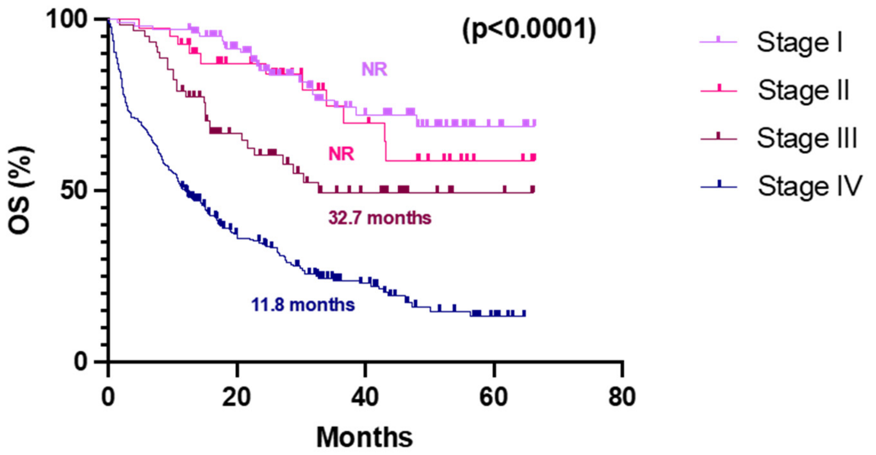

Figure S2. Driver molecular characteristics of all populations.

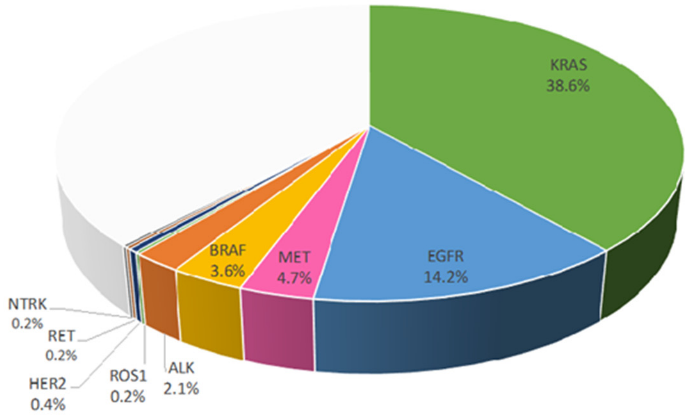

Supplement: Supplementary file 1 [file jcm-14-05135-s001.zip › jcm-3734914-supplementary.pdf]
